# Editorial: Opportunity costs in health care: cost-effectiveness thresholds and beyond

**DOI:** 10.3389/frhs.2023.1293592

**Published:** 2023-10-26

**Authors:** Chris Sampson, Ashley Leech, Borja Garcia-Lorenzo

**Affiliations:** ^1^ Office of Health Economics (OHE), London, United Kingdom; ^2^ Department of Health Policy, Vanderbilt University School of Medicine, Nashville, TN, United States; ^3^ Biosistemak Institute for Health Systems Research, Barakaldo, Spain

**Keywords:** opportunity cost, cost-effectiveness, health technology assessment, marginal productivity, priority-setting, economics

**Editorial on the Research Topic** Opportunity costs in health care: cost-effectiveness thresholds and beyond

Opportunity cost is a contender for the defining concept of economics, so we might expect researchers' and decision-makers' understanding of it to be clear-cut. The four articles in this Research Topic demonstrate that, in the delivery of health services, opportunity cost remains a slippery concept that can be operationalised for policymaking in numerous ways.

One popular approach to thinking about opportunity cost is the cost-effectiveness bookshelf analogy. In this analogy, books represent health technologies, and the opportunity cost of adopting a new technology is represented by the best book that no longer fits on the shelf. Charlton uses this analogy to explore the fairness of restricting access to health care based on its budget impact. A standard illustration of the bookshelf analogy demonstrates that a high budget impact technology has a higher opportunity cost; maintaining a fixed cost-effectiveness threshold could result in the technology displacing more health than it generates. But Charlton shows us that if health care investments and adoption are divisible, then the supposed link between budget impact and opportunity cost falls away. Ripping books from pages changes how we think about the opportunity cost of investing in new technologies.

Another way of thinking about opportunity costs is in relation to the distribution of economic surplus arising from innovations; what proportion of the value (and cost) is borne by consumers, and what proportion by producers? Berdud et al. describe and interrogate a theoretical framework for identifying a cost-effectiveness threshold in the context of a fair distribution of economic surplus. The novelty of their contribution lies primarily in the incorporation of bargaining power. One important finding is that superior outcomes may be achieved by adopting a cost-effectiveness threshold that is higher than typical estimates of opportunity costs based on the current productivity of the health system. If decision-makers seek to maximise the value created by new investments, a complex interpretation of the opportunity cost of decisions may be needed.

Though still grounded in foundational economic theory, a great deal of recent work on this topic has focused on attempts to quantitatively estimate the marginal productivity of expenditure of a health system, which may be used as an indicator of the opportunity cost of new expenditure. Zamora and Towse review quantitative analyses that have sought to estimate the opportunity cost of expenditure in England's National Health Service (NHS). Their objective is to assess the extent and importance of structural uncertainty in these studies, which have typically argued that the cost-effectiveness threshold adopted by the National Institute for Health and Care Excellence (NICE) is too high. The authors argue that due consideration of structural uncertainty may undermine this claim somewhat, with plausible structural assumptions implying that NICE's £20,000–£30,000 per QALY is a legitimate estimate.

The issues discussed in these articles do not routinely make it into the public zeitgeist, but the COVID-19 pandemic changed that as politicians and commentators debated the value of new investments and the trade-offs inherent in different policy decisions. Tinghög and Strand conducted an online survey in August and September 2020 to understand how the public in Sweden felt about priority setting and the use of cost-effectiveness to inform decisions. Generally speaking, respondents preferred health care professionals to be responsible for priority-setting rather than economists, and the notion of basing decisions on opportunity costs was not popular.

The papers in this Research Topic help to shed light on some of the challenging nuances associated with estimating opportunity costs in health care. Whether in theory, in quantitative methodologies, or in public opinion, the role of opportunity cost in decision-making remains contentious and ambiguous.

